# Assimilating South African medical students trained in Cuba into the South African medical education system: reflections from an identity perspective

**DOI:** 10.1186/s12909-016-0800-4

**Published:** 2016-10-24

**Authors:** B. M. Donda, R. J. Hift, V. S. Singaram

**Affiliations:** Clinical and Professional Practice, School of Clinical Medicine, University of KwaZulu-Natal, Private Bag X 7, Congella, 4013 Durban, South Africa

**Keywords:** Discrepancy theory, Identity, Identity crisis, Medical education, Nelson Mandela Fidel Castro Medical Collaboration Programme

## Abstract

**Background:**

In terms of the Nelson Mandela Fidel Castro Medical Collaboration programme, an agreement between the governments of South Africa and Cuba, cohorts of South African students receive their initial five years medical training at a Cuban university before returning to South Africa for a six to twelve months orientation before integration into the local final year class. It is common for these students to experience academic difficulty on their return. Frequently this is viewed merely as a matter of a knowledge deficit.

**Discussion:**

We argue that the problem arises from a fundamental divergence in the outcomes of the Cuban and South African medical curricula, each of which is designed with a particular healthcare system in mind. Using the discrepancy theory of identity proposed by Higgins in 1987, we discuss the challenges experienced by the returning Nelson Mandela Fidel Castro Medical Collaboration students in terms of a potential crisis of identity and suggest interventions which may prove valuable in promoting academic success and successful integration.

**Conclusions:**

Though providing additional training to address the gap in skills and knowledge in returning students is an important part of their successful reintegration, this could be insufficient on its own and must be complemented by a range of measures designed to ameliorate the discrepancies in identity which arise from the transition from one educational model to another.

## Background

South Africa has a severe shortage of health professionals. The Human Resources for Health Plan of 2011 reported that the country had 5.43 doctors per 10 000 population, contrasting poorly with other emerging economies such as Brazil (17.31) and Argentina (31.96) [1]. It suggested an existing deficit of 11,735 medical practitioners and specialists in South Africa, and indicated that South Africa would have to double its production of doctors to close the gap [1, 2]. One method of augmenting physician numbers is to train students abroad, and in 1996 the South African government entered into a bilateral agreement with the government of the Republic of Cuba to establish the Nelson Mandela-Fidel Castro Medical Collaboration programme (NMFCMC), in terms of which selected South African students are sent to Cuba to receive their initial medical training in a Cuban university. All costs of the programme are borne by the South African government including tuition, travel, accommodation and subsistence. Students receive an initial five years training in medicine in Cuba, in Spanish, and then complete their final year in English at South African medical school. Most medical schools require the returning students to join a 6- or 12-month orientation programme before entering the final year. The design, duration and a need for a formal orientation programme is left to the discretion of each university as this exercise is not part of pass requirements but a platform for preparation of such students for final year in the South African context. Basically during orientation student’s s are taught and mentored in the skills lab, ward rounds and sit for mock exams in different disciplines. However, students on the NMFCMC programme are required to pass all the final exit examinations of the South African medical school, alongside locally-trained students, as well as the final Cuban licensing examination (again in Spanish). Having done so, they may enter the first internship year.

Experience has shown that most NMFCMC students experience academic difficulties on their return to South Africa. In the final examinations [3]) approximately (50 %) will have to repeat modules in order to qualify, resulting in a prolongation of training which in some cases may be substantial. Typically the academic problems are seen as a matter of teaching and learning; and there has been a tendency on the part of some academics to view the Cuban programme pejoratively as representing a lower standard of education. In practice assimilation of NMFCMC students is traditional [4] as it involves conforming to SA curative based protocols, ways of thinking and acting without any platform for assessment, usage and learning form the strengths they bring form their Cuban exposure. Therefore interactions with peers and academics (*significant others*) have the potential to shape how NMFCMC students perceive who they are in terms of their roles as medical students and future doctors in SA. We believe that a divergence in the outcomes set for the Cuban and South African curricula which is not necessarily an issue of standards is the main challenge in the NMFCMC programme. Though this may present superficially as a deficit in knowledge and skills at the time of return, we suggest that the consequences may be far-reaching, and may be understood using Higgins’s *discrepancy theory* of identity [5].

## Discussion

### The concept of identity

**Fig. 1:**
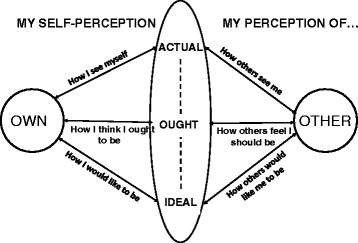
A modified schematic representation of the theory of identity proposed by Higgins [5]

Drawing mainly from James’ work on self and affect published in 1948 [6], Higgins identifies three domains of the self: *actual* (how individuals view themselves), *ought* (how individuals believe they should be) and *ideal* (how individuals wish themselves to be). Each of these may be qualified as *own* (self-perceived identity) and *other* (how individuals perceive themselves to be viewed by others). This is summarised diagrammatically in Fig. 1.

The individual’s concept of self, or identity, then arises from unique configurations of associations between these three domains, and between the *own* and *other* perceptions. The configurations of *ought/own* and *ideal/own* (how I believe I should be and how I would like to be) act as regulatory standards of the *actual/own* (how I am) and may motivate particular behaviours [5, 6]. Significant discrepancies between the individual’s perceptions of who they are and of what they believe to be the perceptions of others constitute an *identity crisis* [7–9]. Erickson defined an identity crises as an inner conflict resulting from incongruence between who others think an individual is and who the individual regards himself to be which has been associated with distress, fear, anxiety and depression [5, 10–12].

### Divergence in Cuban and South African curricula

South Africa suffers a serious imbalance between doctors employed in urban versus rural areas. It has been estimated that only about 35 of the 1200 medical graduates produced annually will choose a rural career in the long term. [1] Indeed, a major advantage of the NMFCMC program from the South African government perspective is that the Cuban medical curriculum is explicitly primary-health-care-orientated [13]. South Africa’s national health planning is predicated on the centrality of primary, district-based health care, yet South African curricula, despite continual discussion and incremental adjustment over many years, are still heavily weighted towards specialist, curative and urban medicine [14]. The junior doctor in South Africa is expected to possess the skills required to manage in-patients in a district hospital, which in South Africa is defined as a hospital dealing with primary care patients, that is those who may be managed by a generalist medical officer rather than a specialist. In contrast to many other parts of the world however, including Cuba, district hospital practice includes practical obstetrics, general anaesthesia, operative surgery and the diagnosis and management of life-threatening acute disease. The Cuban curriculum on the other hand is designed to graduate a “*basic general doctor after six years, who will staff a family doctor’s office while pursuing a residency (specialist training) in comprehensive general medicine (family medicine)*” [13]. Clinical exposure is therefore directed towards ambulatory care skills, with greater emphasis on care of patients in the clinic or family practice setting and health promotion, and a lower expectation in terms of the complexity of the conditions which students are expected to manage; management of many conditions which fall within the responsibility of the general medical graduate in South Africa are reserved for specialists in the Cuban health care system [13]. There is thus a serious mismatch in the outcomes set for the Cuban and South African curricula.

### Issues of identity with NMFCMC students

The NMFCMC students are further disadvantaged by their lack of familiarity with South African medical education practice, such as the centrality of clinical bedside work in training and the use of multiple-choice question (MCQ) examinations, objective structured clinical examinations (OSCE) and bedside clinical examinations in assessment, activities of which they typically have had little previous experience. To date there has been no published study that explores strengths of the returning NMFCMC medics. However, recent work has shown that since Clinical training in Cuba occurs in hospital wards while local students first train in a clinical skills laboratory prior to hospital exposures, returning NMFCMC students self-reported a lack of familiarity and confidence to perform procedures with many of the practical procedures expected of locally-trained students by the end of their fourth year [15]. Thus the students are unable to perform as well as locally-trained students in the course of day-to-day clinical teaching as well as formal assessment. This sets up a divergence in how the student sees themselves and how they are perceived by others, leading to serious discrepancies *in actual*/*ought* and *own/other* identity. This provides the ideal catalyst for the crisis of identity described by Higgins [5] and Erickson [7].

NMFCMC students are strongly identified as South African students while in Cuba, yet on their return to a South African university they find themselves labelled as ‘Cuban’ students. Though unintentional in most cases, this labelling serves to identify these students, who view themselves as South African, as alien, leading to a lack of congruence of *actual/own* and *actual/other* identity. Furthermore, it is not uncommon for academics and students to label the NMFCMC students as ‘Cuban’ in a pejorative sense, because of perceived shortcomings in their academic performance or on the basis of personal disagreement with the political principles underlying the NMFCMC programme, thus adding an *ought*/*other* dimension to the problem of identity. The consequence is a marked lack of congruence of these various dimensions of identity.

### Consequences of the discrepancy in identity

A lack of confidence in performance results in fear, vulnerability, unworthiness, embarrassment and loss of face in front of the locally-trained students, and the discrepancy between the students’ self-perceptions (*actual/own*) and their perception of how they are viewed by their South African-trained peers and teachers (*actual/other)* and (*ought/other)*, leads to a widening gap between *actual/own, ought/own* and *ideal/own identities.* Repeated mistakes are likely to elicit a pattern of negative responses from peers and teachers which in turn leads to resentment, fear of criticism and social interaction anxiety [16, 17]. This may lead to an avoidance of situations in which their performance is likely to be shown up in comparison with their local colleagues and a reluctance to interact with other students professionally which may extend to social interactions outside the learning environment; resulting in a tendency for NMFCMC students to retreat into their own social space, thus further isolating themselves and heightening the sense of otherness [5]. This has been described by Johns and Peters as *loss of social affection* [16].

Comparison of the *actual/own* with *ideal/own* may lead to guilt, low self-esteem and unhappiness, potentially leading to depression [16–18]. Clearly this is likely to establish a self-perpetuating vicious cycle of under-performance, loss of confidence and withdrawal from learning opportunities. Self-esteem is critical: though it may be seen as an *outcome* of the congruence of *actual* and *ought* self, it is also a *resource* that protects individual against the effects of discrepancies and a *goal* that motivates how people act [19, 20]. The academic transcripts we receive from the Cuban universities indicate solid performance and thus we assume that NMFCMC students have appropriate confidence and self-esteem when they join us. Though this may be viewed in one sense as positive, it also magnifies the negative consequences of the sudden change in expectations. Individuals with high self-esteem tend to value self-accountability and are therefore prone to become intensely anxious in the face of *actual/ought* discrepancies; they are therefore more vulnerable to psychological harm where their performance fails to match their expectations [20].

### Essential steps in ameliorating the negative consequences of discrepant identity

We believe that educational interventions solely designed to allow the NMFCMC students to catch up” with their local counterparts, though a necessary response, are insufficient. We believe that explicit acknowledgement of the issues of identity which arise in the students allows the institution to work actively to minimise the discrepancies in *actual*, *ideal* and *ought* identity, both *own* and *other.* Recommendations arising from the regulatory focus theory (RFT) of Higgins et al. [21] are summarised and adapted in Table 1. Higgins based RFT on the hedonic principle that suggest that individuals are motivated by an inclination to avoid negative outcomes (prevention) and the adoption of behaviours which promote positive outcomes (promotion). Hence, prevention regulatory strategies in the NMFCMC involve vigilance through regulations and the promotion strategies involve eagerness for student development and growth.

**Table 1:** Strategies to ameliorate identity discrepancy in the returning NMFCMC students (Adapted From Higgins [5]

Narrow the gap between the student’s self-concept and the regulatory self-concepts (ought/ own and/or ideal/own) by:
1. Facilitating an improvement in the student’s performance. * • Additional instruction and training provided in a positive and supportive manner.*
2. Modifying the student’s interpretation of their own performance. • Continually reinforce the valuing of the student’s personal strengths, and encourage an understanding of the reason why current performance may appear inferior without this reflecting on the student’s actual abilities or worth.
3. Protecting the student from circumstances and social interactions which widen the gap between identities. * • Avoid teaching, instruction and testing in mixed groups until such time as the skills and knowledge gap has been significantly narrowed.*
4. Encouraging the student to rehearse and evince positive attitudes actively. * • Use of mentors to close the gap in a positive and non-threatening fashion* * • Group discussion and reflection, particularly with senior staff and role models*
5. Promoting good relationships * • Mutual understanding and a sympathetic, non-judgmental appreciation of the challenges faced by NMFCMC student on the part of local staff and students* * • Promote social interaction under non-threatening conditions which reinforce a shared identity rather than one of a local-foreign divide*
6. Moderating the tendency of local academics and students to widen the actual, ought and ideal discrepancies as the agents of the *other* identity. * • Discourage the term “Cuban student” and “Cuban programme”* * • Forbid all forms of disparagement and statements which are likely to result in a negative self-image on the part of the NMFCMC or to reinforce negative stereotypes on the part of local staff and students.* * • Encourage expressions of positive appreciation of the values imparted by the Cuban curriculum* * • Sensitize staff and students to the negative consequences (even where unintended) of comparisons of ability between local and NMFCMC students in domains in which active remediation has not yet been provided.*

### Self-conceptualisation

The intention is to change the student’s *actual/own* identity to become less incongruent with the *ought/own* and *ideal/own* identities. In our introductory programme we address issues of identity explicitly. Regular group meetings with carefully selected staff and the Dean reinforce self-esteem by affirming their worth as valued students and future doctors and provide a safe space in which they may express some of the identity issues they encounter. They are also invited to participate in artistic activities such as the preparation of collages and poetry which allow them to express who they believe they are as a doctor, and to reflect on past experiences that have shaped them as individuals while giving them the opportunity to express “reasons, meanings and emotions” related to how they see themselves currently [22].

#### Improve performance

It is essential to close the knowledge gap. The more the skills and knowledge converge with those of the locally-trained students, the less apparent the difference between them and therefore the more convergent are the *actual* and *ought* identities. We have an educationally-trained academic whose full-time responsibility is the NMFCMC programme and who is central to aligning the teaching of our academic staff with the needs of the students. We employ academic development officers who monitor students’ progress, offer personalized support and training in study skills, organised peer mentorship and coordinate additional academic support and support from the counselling service. Senior, academically strong and appropriately motivated students are assigned as peer mentors; in practice we find that they tend to operate as student tutors, providing the NMFCMC students with personal assistance in developing appropriate knowledge and skills. We believe that targeted assistance clarifies set expectations and how to achieve them, thus reducing the chance of failure and improve the chance of success in situations which invite potentially unfavourable comparison with locally-trained students. We are aware that enrolling NMFCMC students in exclusive programmes could perpetrate further unintended segregation.

#### Modify students’ interpretation of their performance

The more consciously aware the individual is of their *ought* and *ideal* goals—termed *goal strength*—the more able they are to moderate the adverse effects of non-congruent identities [21, 23]. Thus, where these goals are *cognitively accessible*, the student is enabled to deal with them positively. We use the Metacognitive Awareness Inventory [24] and the Biggs Revised Two Factor Study Questionnaire [25] to increase the students’ conscious awareness of their study habits. Thus NMFCMC students are explicitly made aware of the *ought* and *ideal* goals necessary to moderate discrepancy between their configurations of the self.

Higgins proposed that modification of the individual’s interpretation of their own performance may moderate self-concept and minimise identity crises, and refers to this as *moderating persistent behaviour* [5]. Frank discussion of their performance in a safe environment reinforces the NMFCMC students’ understanding that “they” are not the problem; the problem is a legitimate and foreseeable consequence of their training in a system with legitimate expectations which differ from those of the South African system, and that the discrepancies can and will be overcome. They are also encouraged to reflect on the strategies which led to success while in Cuba, and how these might be modified to result in similar success in the South African context.

#### Reduce exposure to circumstances and social interactions that promote self-discrepancies

A common debate in South African medical schools is whether NMFCMC students should be trained as a separate group or be integrated in to study groups with locally-trained students. Some individuals frequently recommend that they are fully integrated with the locally-trained students, in order to promote a sense of shared identity and therefore assimilation. Currently, traditional assimilation allows space for harbouring individual prejudices against Cuba that attribute to NMFCMC students struggle to integrate. In practice, training them alongside locally-trained students places them at continual risk of unfavourable comparison between their own performance and that of locally-trained students, with all the consequences described earlier in this paper. Self-discrepancy theory recommends *reduction* of social interaction and activities that widen self-discrepancies [5]. Our own experience suggests that they benefit from being trained as a separate, homogeneous group. In terms of Bamberg’s model, the transition to the South African model of learning and practice can be explicitly managed by a team sensitive to issues of identity and self-esteem and aware of their specific learning needs including practice in local assessment methodologies, and their sense of agency can be maintained as they adapt to new requirements. Setting achievable goals and progressively aligning these with those expected of locally-trained students narrows the *actual/ought* discrepancy. They are protected against unfavourable comparison with locally-trained students, thus, reducing social interaction anxiety. Once the performance gap has been narrowed, they are better able to assimilate into a mixed class, and determining when students are ready for this, as opposed to setting an arbitrary time for integration, is important.

#### Mentoring

Those of our NMFCMC students who perform most competently and confidently frequently ascribe their success to their having found a suitable mentor in the clinical area. Typically the student and one of the ward staff have providentially struck up an informal mentoring relationship. This correlation is almost certainly not coincidental: Turban and Dougherty [26] have indicated that individuals with good internal control, metacognitive awareness and emotional stability, actively seek a mentoring relationship and are likely to make it work once they find one. The benefits of mentoring extend beyond the actual knowledge and skills imparted by the mentor to the student. Mentoring has a major positive benefit in the rebalancing and validation of identities. This is followed by a virtuous cycle of increasing confidence, knowledge and performance. Furthermore, the mentoring relationship affirms the uniqueness of the student’s identity, and since the agenda of the relationship is set by the student, their sense of agency too is reinforced.

We believe that assigning mentors formally to students may not, in terms of identity theory, always be successful and may even be harmful. It is the *spontaneous* demonstration of interest in the student on the part of the mentor that is critical to the success of the relationship. As a specific intervention for the NMFCMC students, formally assigning them to mentors may potentially be counter-productive in terms of reinforcing the sense of otherness and of inferiority; with a loss of the sense of agency, the mentor being seen to be there to “tell them what to learn, what to do and how to fit in”.

#### Promoting constructive relationships with peers and teachers

The role of the local academics and students in the formation of the *actual/other*, *ought/other* and *ideal/other* identities cannot be underestimated, as these are perhaps the most significant factor in generating identity discrepancies. It is essential that locally-trained students and staff are discouraged from referring to the NMFCMC students as “Cuban students”, thus reinforcing their otherness. We use a neutral term, “collaboration students”. We continually reinforce the notion that the contribution of the NMFCMC students following graduation is critically needed in South Africa, both in terms of their augmentation of the human resource base for our health care system, and in terms of the primary health care approach they bring to bear on local health challenges. Secondly we remind our staff at all opportunities that the NMFCMC students are trained to a *different* set of outcomes, rather than to an *inferior* standard. One returning NMFCMC student has written movingly of the hurt of hearing themselves referred to as “useless returnees” by lecturers in conversation with the locally-trained students [27]. It is critical that no impression is given that the NMFCMC students are seen as a problem or an inconvenience, or that the value of their programme is questioned.

According to Maslow’s theory of needs, lack of interaction, human relationships and the sense of belonging may result in depression or loneliness while an abundance of love and community often sustain people through difficult times [28]. Taking active steps to prevent social isolation is important. Wilcox, Winn [29] have shown that living arrangements wherein new students lived alongside more senior students were central in enabling a form of emotional support analogous to that offered by families and thus in moderating the effects of stressful situations. We have observed that NMFCMC students who reside in student residences experience a greater degree of social support from local students and tend to perform more ably. Here the relationship is essentially equal, supportive and collaborative, since the students are meeting in a neutral space, and the element of comparison which characterises the relationship in the learning environment is minimised.

#### Congruence of goals

From our experience the *ought* and *ideal* self-discrepancies are the most difficult to deal with in the NMFCMC programme. Established *ideal* and *ought* selves remain fairly stable in the face of all other changes. Students are typically socialised by their seniors into standard *actual* identities and are regulated by a form of socially-mediated accountability when they fail to conform. [30] The Cuban health care model has been very successful in that Cuban health indicators are among the highest in the world, and the Cuban education model is designed to graduate a particular type of doctor suitable for deployment in that system. The South African government professes to aspire to a health care system analogous to that of Cuba. Yet no allowance, consideration or credit is given to the NMFCMC students on their return for their proficiency when acting within the primary health care model. Rezler [31] concluded from a review of the literature that attitudes learned in one setting were not carried over to another setting where they are not acknowledged or prioritised. Becker [32], in a study examining the impact of training medical undergraduates observed that where students’ values were regarded as irrelevant by their peers and teachers, students often subsequently failed to act in accordance with those values even in circumstances where they would have been appropriate.

The NMFCMC students are held to the same competencies as locally-trained students, are therefore frequently tested on knowledge and skills which were never stressed in their training and via assessment methodologies with which they are unfamiliar. In practice they are essentially not tested against the outcomes for which they were previously trained. This is a classic example of being set up for failure, and is the origin of much of the serious discrepancies in identity and apparently poor performance which are encountered in the NMFCMC returnees. Some honest reflection on the part of South African academics might suggest that the fault should not be ascribed to the Cuban education educational model, but at least in part to the heavy emphasis on hospital-centred curative care in South African curricula which persists despite years of talk of making it more relevant to needs at community level. Curriculum reforms in South African universities geared towards a PHC orientation are fledgling. Only then could students trained in the South African system be required to demonstrate more of the skills stressed in the Cuban curriculum, thus the reduction of the apparent gap in performance. In identity theory terms, this is equivalent to changing the self-regulatory concepts *ought* and *ideal* to match the *actual/own* identity of the returning NMFCMC student more closely. The current authors have seen a need to conduct a qualitative study focusing on the socialisation of the NMFCMC in a South African university.

## Conclusions

In conclusion, we believe that issues of identity are central to the academic difficulties evident in our returning NMFCMC students. Explicit recognition of these will suggest effective responses to minimise the difficulties experienced by the students, promote academic success, facilitate their integration into the South African health care system and maximise their eventual contribution to the solution of the health challenges facing our country. The size of the NMFCMC programme has increased in recent years, and more than 800 students will be returning to South Africa annually from 2018. This will pose problems of scale which require resourceful and innovative responses. Furthermore, a typical ratio of applicants for admission to South African medical schools and places on offer, as observed in our institution, is 14:1. Consequently large numbers of South African students who cannot be accommodated locally, have enrolled for medical training in other countries, such as China, where the medical educational model is very different to that followed in South Africa. These students potentially provide a valuable resource to the country, particularly as most of them have trained at no expense to the state, whereas medical training for local students is heavily subsidised. Yet as we have seen from the NMFCMC experience, reintegration will not be easy. Though the detail will differ, the fundamental identity issues described in our paper will form a point of commonality with our experience in the NMFCMC students, and similar principles may be applied to meeting the challenge.

## Abbreviations

NMFCMC: Nelson Mandela Fidel Castro Medical Collaboration
